# Comparison of Efficacy of Atorvastatin and Rosuvastatin in Patients With Acute Coronary Syndrome: A Systematic Review and Meta-Analysis

**DOI:** 10.7759/cureus.68602

**Published:** 2024-09-04

**Authors:** Darab Shuja, Muhammad Umar Mian, Manpreet Kaur Dhanjal, Jaina Mengar, Aqsa A Butt, Sandipkumar S Chaudhari, Calvin R Wei, Areeba Khan

**Affiliations:** 1 Internal Medicine, Services Hospital Lahore, Lahore, PAK; 2 Internal Medicine, Allama Iqbal Medical College, Lahore, PAK; 3 Medicine, Adesh Institute of Medical Sciences and Research, Ludhiana, IND; 4 Medicine and Surgery, Government Medical College and New Civil Hospital, Surat, IND; 5 Internal Medicine, Allama Iqbal Medical College, lahore, PAK; 6 Cardiothoracic Surgery, University of Alabama at Birmingham, Birmingham, USA; 7 Family Medicine, University of North Dakota School of Medicine and Health Sciences, Fargo, USA; 8 Research and Development, Shing Huei Group, Taipei, TWN; 9 Critical Care Medicine, United Medical and Dental College, Karachi, PAK

**Keywords:** systematic review and meta-analysis, mace, acute coronary syndrome, rosuvastatin, atorvastatin

## Abstract

Acute coronary syndrome (ACS) remains a leading cause of morbidity and mortality worldwide. Statins, particularly atorvastatin, and rosuvastatin, are crucial in managing cholesterol levels and reducing cardiovascular risk in ACS patients. However, direct comparative studies between these two statins are limited. This meta-analysis aimed to compare the efficacy of atorvastatin and rosuvastatin in reducing major adverse cardiovascular events (MACE) and all-cause mortality in patients with ACS. A comprehensive literature search was conducted in PubMed, Embase, Cochrane Library, and Scopus for studies published up to July 2024. Randomized controlled trials and observational studies directly comparing atorvastatin and rosuvastatin in ACS patients were included. The primary outcomes were the incidence of MACE and all-cause mortality. Six studies involving 4195 patients were included in the meta-analysis. Pooled analysis showed no statistically significant difference between atorvastatin and rosuvastatin in reducing MACE [risk ratio (RR): 0.91, 95% confidence interval (CI): 0.68 to 1.22, p-value: 0.54] or all-cause mortality (RR: 0.94, 95% CI: 0.52 to 1.70, p-value: 0.83). No significant heterogeneity was observed among the studies (I-square: 0% for both outcomes). This meta-analysis suggests that atorvastatin and rosuvastatin have comparable efficacy in reducing MACE and all-cause mortality in ACS patients. These findings provide clinicians with flexibility in choosing between these statins based on individual patient factors. However, further large-scale randomized controlled trials are needed to confirm these results and explore potential differences in specific patient subgroups.

## Introduction and background

Globally, acute coronary syndrome (ACS) and other forms of ischaemic heart disease are the primary causes of morbidity and death. ACS is a common health problem worldwide and is estimated to affect more than seven million people each year [1]. Statistics from the World Health Organisation show that in 2016, it was responsible for almost nine million deaths worldwide [2]. The burden of ACS is expected to rise due to the increasing prevalence of risk factors such as hypertension, diabetes, obesity, and aging populations [3]. Advances in medical therapies and interventions have improved outcomes for many patients, but the disease remains a major public health challenge. Preventative measures, early diagnosis, and effective management are critical to reducing the global impact of ischemic heart disease [4]. 

To manage cholesterol levels in the context of primary and secondary prevention of coronary artery disease (CAD), statins are frequently administered. Statins hold an IA class of recommendation (CoR) in major guidelines for patients with ACS, including those with ST-elevation myocardial infarction (STEMI) and non-ST-elevation ACS (NSTE-ACS). These patients must begin taking statins as soon as possible, provided there are no contraindications and regardless of their baseline cholesterol levels at the time of the acute event [5-6]. Atorvastatin and rosuvastatin, both belonging to the 3-hydroxy-3-methylglutaryl coenzyme A (HMG-CoA) reductase inhibitor class, are frequently prescribed statins. These medications play a crucial role in controlling elevated cholesterol levels, thereby mitigating the risk of cardiovascular diseases. Atorvastatin is widely acknowledged for its effectiveness in significantly lowering low-density lipoprotein cholesterol (LDL-C) [7]. It is often prescribed due to its proven track record in reducing the incidence of heart attacks and strokes. On the other hand, rosuvastatin is commended for its high potency, enabling it to achieve substantial reductions in LDL-C even at lower doses compared to other statins [7]. Additionally, rosuvastatin has a notable impact on raising high-density lipoprotein (HDL) cholesterol, which is beneficial for cardiovascular health. While both statins are effective, the choice between atorvastatin and rosuvastatin may depend on individual patient profiles, specific cholesterol targets, and the presence of any underlying conditions. Clinical studies suggest that rosuvastatin may offer superior LDL-C lowering capabilities, but atorvastatin remains a highly effective and widely used option in lipid management [8]. 

Previous studies have predominantly compared the efficacy of atorvastatin or rosuvastatin against placebo, demonstrating their significant benefits in lowering LDL-C and reducing cardiovascular events [9]. However, there is a notable paucity of direct comparative studies between atorvastatin and rosuvastatin. The few existing studies that do compare these two statins often involve limited sample sizes, reducing the robustness and generalizability of their findings. This lack of comprehensive comparative data creates uncertainty in clinical decision-making regarding which statin may be more effective in reducing major adverse cardiovascular events (MACE) in patients with aACS. This study aims to compare the efficacy of atorvastatin and rosuvastatin in reducing MACE in patients with ACS. By directly comparing these two commonly prescribed statins, this study seeks to provide evidence-based guidance for clinicians in selecting the most effective lipid-lowering therapy for secondary prevention in ACS patients. 

## Review

Methodology 

This methodology ensures a rigorous and systematic approach to comparing atorvastatin and rosuvastatin in reducing MACE in ACS patients, adhering to Preferred Reporting of Systematic Review and Meta-analysis (PRISMA) guidelines. 

Literature Search 

We conducted a comprehensive literature search to identify relevant studies comparing the efficacy of atorvastatin and rosuvastatin in reducing MACE in patients with ACS. The search was performed in multiple databases, including PubMed, Embase, Cochrane Library, and Scopus. We included studies published up to July 2024. We used the following search terms and their combinations: “atorvastatin,” “rosuvastatin,” “acute coronary syndrome,” “ACS,” “major adverse cardiovascular events,” “MACE,” and “statins.” No restriction was placed on the time and language of publication. We also manually screened the reference lists of selected articles and relevant reviews to ensure a comprehensive search. The search was performed by two authors independently. Any disagreement between the two authors was resolved through discussion. 

Study Selection 

Studies were selected based on predefined inclusion and exclusion criteria. We included randomized controlled trials (RCTs) and observational studies that directly compared atorvastatin and rosuvastatin in patients diagnosed with ACS. Studies were required to report on MACE or mortality as an outcome. We excluded studies that compared either statin to a placebo or other interventions, studies that did not report on MACE, and studies that enrolled patients without ACS. Two independent reviewers screened titles and abstracts for relevance, followed by a full-text review to confirm eligibility. Discrepancies were resolved through discussion or consultation with a third reviewer. 

Data Extraction and Quality Assessment

Data extraction was performed independently by two reviewers using a standardized data extraction form developed on Microsoft Excel (Microsoft Corporation. (2018). Microsoft Excel). Extracted data included study characteristics (author, year, study design, sample size, duration of follow-up), patient characteristics (age, sex, diabetes, and hypertension), intervention details (dosage of atorvastatin and rosuvastatin), and outcomes (incidence of MACE, and all-cause mortality). Any disagreements were resolved by discussion or by consulting a third reviewer. Quality assessment of included studies was assessed using the Cochrane Risk of Bias assessment tool and New-Castle Ottawa Scale for RCTs and observational studies respectively. 

Data Synthesis and Analysis 

We conducted a meta-analysis using Review Manager (RevMan) software (2020, The Cochrane Collaboration, Copenhagen). The primary outcome was the incidence of MACE and all-cause mortality in patients treated with atorvastatin versus rosuvastatin. We calculated pooled risk ratios (RRs) and 95% confidence intervals (CIs) using a random-effects model to account for variability among studies. Heterogeneity was assessed using the I² statistic, with values above 50% indicating substantial heterogeneity. 

Results 

**Figure 1:**
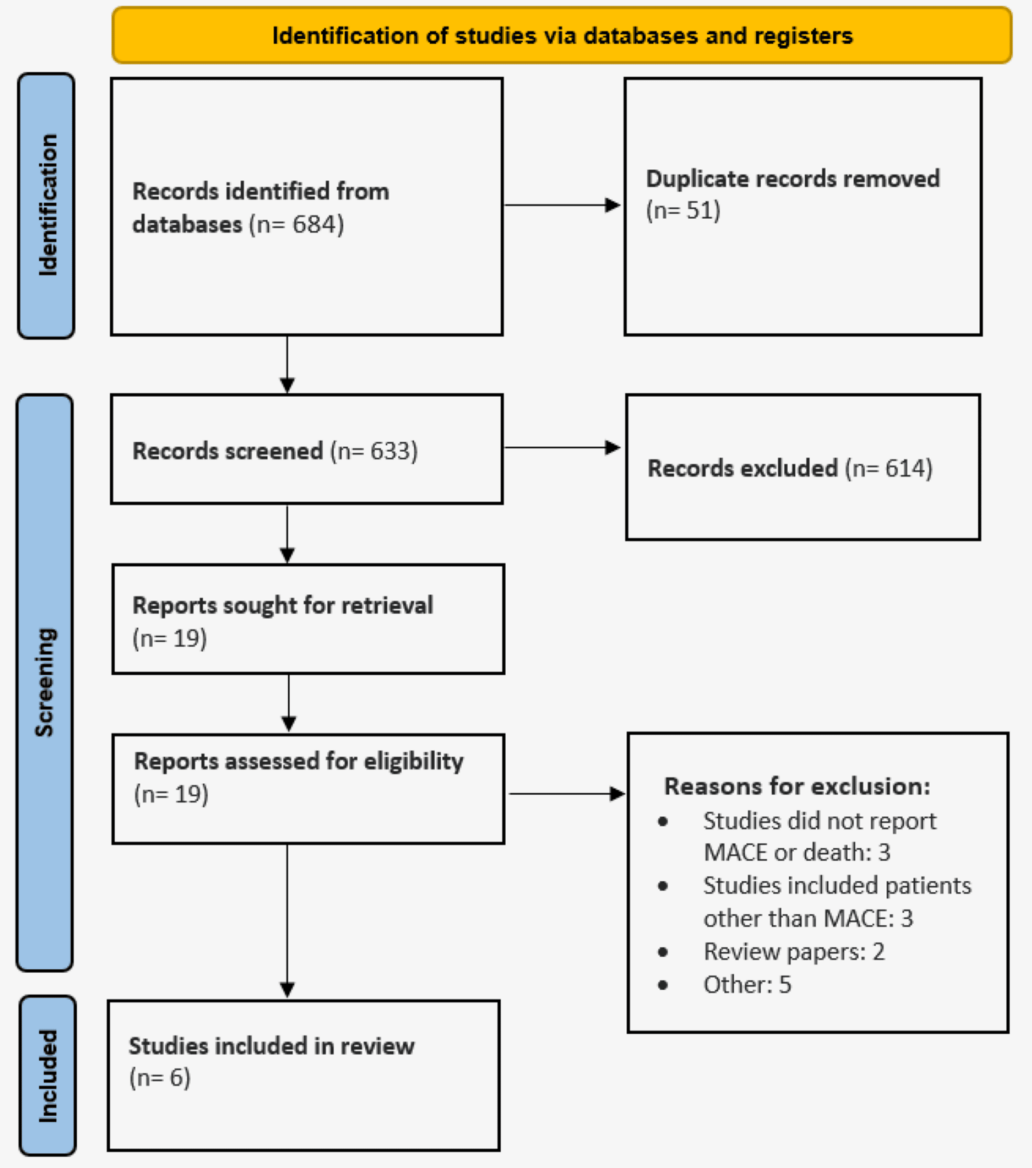
PRISMA flowchart demonstrating the study selection process

Six hundred and eighty-four records in all were located using electronic databases. Six hundred and thrity-three records were checked for eligibility after duplicates were eliminated, and 614 of them were deemed irrelevant. After obtaining the whole texts of all 19 records, we evaluated each record's eligibility using pre-established inclusion and exclusion criteria. As a result, this review contains six studies. The revised PRISMA 2020 statement can be found in Figure 1, which displays the study selection flowchart. The features of the included studies are displayed in Table 1. Three of the six studies were retrospective, and three were RCTs. Table 2 presents the characteristics of patients enrolled in individual studies. Table 3 presents a quality assessment of the included studies

**Table 1:** Characteristics of included studies RCT: randomized-control trial

Author	Year	Study Design	Region	Follow-up	Study Dose		Sample Size	
					Atorvastatin	Rosuvastatin	Atorvastatin	Rosuvastatin
Dai et al. [10]	2020	Retrospective	China	In hospital	20 mg	10 mg	585	195
He et al. (a) [11]	2022	RCT	China	1 Week	80 mg	20 mg	31	34
He et al. (b) [11]	2022	RCT	China	1 Week	40 mg	10 mg	33	32
Lablanchea et al. [12]	2010	RCT	Multinational	12 Weeks	80 mg	20 mg	450	437
Pitt et al. [13]	2012	RCT	United States	In hospital	80 mg	40 mg	278	270
Rahhal et al. [14]	2022	Retrospective	Qatar	52 Weeks	80 mg	20 to 40 mg	626	627
Zhou et al. [15]	2023	Retrospective	China	In hospital	20 mg	10 mg	415	182

**Table 2:** Characteristics of subjects

Author	Groups	Sample size	Mean age	Males (n)	Hypertension (n)	Diabetes (n)
Dai et al. [10]	Atorvastatin	585	62.92	472	303	156
	Rosuvastatin	195	62.74	161	103	49
He et al. (a) [11]	Atorvastatin	31	68.6	17	10	11
	Rosuvastatin	34	67.2	16	11	10
He et al. (b) [11]	Atorvastatin	33	69.3	18	9	8
	Rosuvastatin	32	68.4	17	8	9
Lablanchea et al. [12]	Atorvastatin	450	59	344	228	82
	Rosuvastatin	437	60	321	235	82
Pitt et al. [13]	Atorvastatin	278	52.9	219	139	35
	Rosuvastatin	270	52.8	200	137	35
Rahhal et al. [14]	Atorvastatin	626	50	606	254	296
	Rosuvastatin	627	52	594	242	286
Zhou et al. [15]	Atorvastatin	415	61.98	341	191	106
	Rosuvastatin	182	60.51	157	89	38

**Table 3:** Quality assessment of included studies

Quality Assessment of Observational Studies						
	Selection	Exposure	Outcome	Overall		
Dai et al. [10]	3	2	3	Good		
Rahhal et al. [14]	3	1	2	Fair		
Zhou et al. [15]	4	2	3	Good		
Quality Assessment of RCTs						
	Selection	Performance	Detection	Attrition bias	Reporting bias	Other Bias
He et al. [11]	Low	Low	Low	Low	Low	Low
Lablanchea et al. [12]	Low	Low	Low	Low	Low	Unclear
Pitt et al. [13]	Low	Low	High	Unclear	Low	Unclear

Comparison of Incidence of MACE between Atorvastatin and Rosuvastatin 

**Figure 2:**
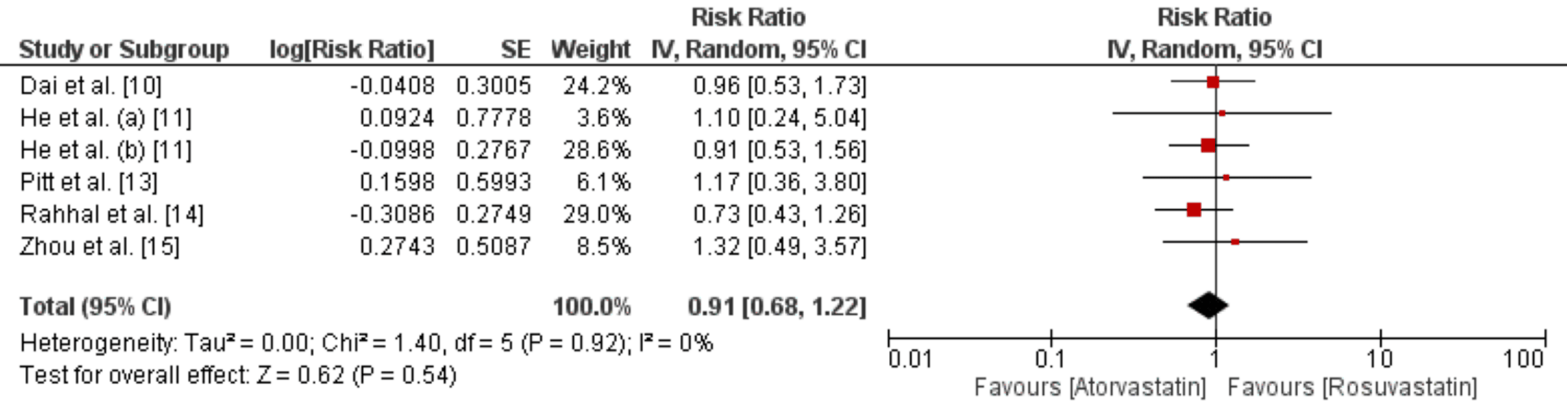
Comparison of incidence of MACE between atorvastatin and rosuvastatin

MACE was compared between two groups and the results are presented in Figure 2. Pooled analysis showed that the risk of MACE was not significantly different between patients who received atorvastatin and rosuvastatin (RR: 0.91, 95% CI: 0.68 to 1.22, p-value: 0.54). In terms of heterogeneity, we did not find any heterogeneity among the study results (I²: 0%). None of the studies showed a significant association of drugs with MACE. 

Comparison of Incidence of All-cause Mortality between Atorvastatin and Rosuvastatin 

**Figure 3:**
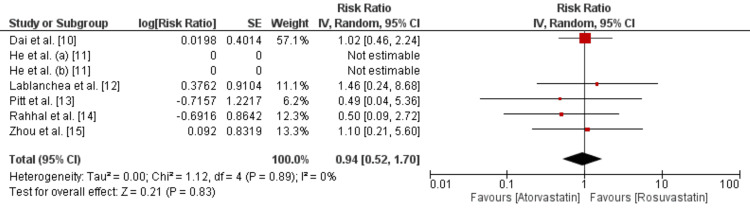
Comparison of incidence of death between atorvastatin and rosuvastatin

All-cause mortality was compared between two groups and the results are presented in Figure 3. Pooled analysis showed that the risk of all-cause mortality was not significantly different between patients who received atorvastatin and rosuvastatin (RR: 0.94, 95% CI: 0.52 to 1.70, p-value: 0.83). In terms of heterogeneity, we did not find any heterogeneity among the study results (I²: 0%). None of the studies showed a significant association of drugs with all-cause mortality.

Discussion 

Data obtained from 4,195 patients across six studies were included in this meta-analysis. The analysis compared the efficacy of atorvastatin and rosuvastatin in reducing major adverse cardiovascular events (MACE) and all-cause mortality in patients with acute coronary syndrome (ACS). The pooled results demonstrated that there was no statistically significant difference between the two statins in terms of reducing MACE. The risk ratios (RRs) for MACE reduction were similar for both atorvastatin and rosuvastatin, indicating that neither drug was superior in preventing these critical events. Importantly, the analysis reported no significant heterogeneity among the study results. The I² statistic was low, indicating consistency in the findings across the included studies. 

Research has also highlighted the impact of different statin doses in myocardial infarction treatment. One study found that high-dose reloading of both rosuvastatin and atorvastatin improved procedural and long-term clinical outcomes in stable patients on chronic statin therapy [16]. Another study reported that early intensive rosuvastatin therapy could enhance clinical outcomes in patients with acute myocardial infarction (AMI) [17]. The observed similarity in efficacy between atorvastatin and rosuvastatin in preventing cardiovascular disease (CVD) can be attributed to their shared mechanism of action and potent cholesterol-lowering abilities [18]. Both drugs belong to the statin class and function by inhibiting HMG-CoA reductase, a key enzyme in the cholesterol synthesis pathway. This inhibition reduces circulating LDL-C levels, which is a major contributor to atherosclerosis and cardiovascular events [19]. 

Wei et al.'s meta-analysis, which was based on four chosen studies, revealed no statistically significant differences in composite cardiovascular events, cardiovascular mortality, myocardial infarction, or stroke between atorvastatin- and rosuvastatin-treated patients [20]. This result demonstrates the same effectiveness of both statins in reducing cardiovascular risks and their clinical equipoise in secondary prevention. 

In conclusion, our meta-analysis offered some evidence in favor of the claim that statins are effective in treating or lowering the risk of MACE. Nevertheless, there isn't enough research, therefore we only examine the RR data. Future large-scale, carefully planned trials may be required, and they should take the interactions between various statins into account. Additionally, further research should explore the potential differential effects of atorvastatin and rosuvastatin on other cardiovascular biomarkers, patient adherence, and cost-effectiveness. Such studies will enhance our understanding of the nuances in statin therapy and support the development of personalized treatment strategies to optimize cardiovascular outcomes in patients with ACS. 

Clinical Implications 

The findings of this meta-analysis have important clinical implications. Given the comparable efficacy of atorvastatin and rosuvastatin in reducing MACE and all-cause mortality, clinicians have flexibility in choosing between these two statins based on individual patient characteristics, tolerability, and potential drug interactions. The decision can also be influenced by factors such as cost, patient preference, and availability of the medication [21-22]. Understanding that both statins are equally effective can help in shared decision-making and tailoring treatment plans to individual patient needs. 

*Study Limitations* 

The present meta-analysis has certain limitations. Firstly, a number of included studies is less and out of these studies, only 3 were RCTs. As observational studies are associated with selection bias, we need future large-scale RCTs to compare the efficacy and safety of these two drugs in ACS patients. Secondly, we were not able to perform subgroup analysis based on the regimen, comorbidities, age, and gender due to the lack of availability of individual-level data. This subgroup analysis is important in future research to identify how these two drugs perform differently in different groups of people. 

## Conclusions

This meta-analysis compared the efficacy of atorvastatin and rosuvastatin in reducing major adverse cardiovascular events (MACE) and all-cause mortality in patients with acute coronary syndrome (ACS). The results showed no statistically significant difference between the two statins in terms of MACE reduction or all-cause mortality. This suggests that both drugs are equally effective in secondary prevention for ACS patients. The findings provide clinicians with flexibility in choosing between these statins based on individual patient factors. However, the study has limitations, including a small number of included studies and the inability to perform subgroup analyses. Future large-scale randomized controlled trials are needed to confirm these findings and explore potential differences in efficacy among specific patient subgroups.
